# Exploring Molecular Mechanism of Huangqi in Treating Heart Failure Using Network Pharmacology

**DOI:** 10.1155/2020/6473745

**Published:** 2020-04-23

**Authors:** Yan-gu Tao, Xiu-Fang Huang, Jun-yan Wang, Meng-ru Kang, Ling-jun Wang, Shao-xiang Xian

**Affiliations:** ^1^The First Affiliated Hospital of Guangzhou University of Chinese Medicine, Guangzhou, China; ^2^Sun Yat-sen Memorial Hospital, Sun Yat-sen University, Guangzhou, China; ^3^Lingnan Medical Research Center, Guangzhou University of Chinese Medicine, Guangzhou, China

## Abstract

Heart failure (HF), a clinical syndrome with a high incidence due to various reasons, is the advanced stage of most cardiovascular diseases. Huangqi is an effective treatment for cardiovascular disease, which has multitarget, multipathway functions. Therefore, we used network pharmacology to explore the molecular mechanism of Huangqi in treating HF. In this study, 21 compounds of Huangqi, which involved 407 targets, were obtained and reconfirmed using TCMSP and PubChem databases. Moreover, we used Cytoscape 3.7.1 to construct compound-target network and screened the top 10 compounds. 378 targets related to HF were obtained from CTD and GeneCards databases and HF-target network was constructed by Cytoscape 3.7.1. The 46 overlapping targets of HF and Huangqi were gotten by Draw Venn Diagram. STRING database was used to set up a protein-protein interaction network, and MCODE module and the top 5 targets with the highest degree for overlapping targets were obtained. GO analysis performed by Metascape indicated that the overlapping targets were mainly enriched in blood vessel development, reactive oxygen species metabolic process, response to wounding, blood circulation, and so on. KEGG analysis analyzed by ClueGO revealed that overlapping targets were mainly enriched in AGE-RAGE signaling pathway in diabetic complications, IL-17 signaling pathway, HIF-1 signaling pathway, c-type lectin receptor signaling pathway, relaxin signaling pathway, and so on. Finally, molecular docking showed that top 10 compounds of Huangqi also had good binding activities to important targets compared with digoxin, which was carried out in CB-Dock molecular docking server. In conclusion, Huangqi has potential effect on regulating overlapping targets and GE-RAGE signaling pathway in diabetic complications, IL-17 signaling pathway, HIF-1 signaling pathway, and so on to be a latent multitarget, multipathway treatment for HF.

## 1. Introduction

Heart failure (HF) is a clinical syndrome of cardiac insufficiency caused by abnormalities in cardiac structure remodeling due to various reasons, which is the advanced stage of most cardiovascular diseases' progression [1]. The prevalence of HF in adults is as high as 0.9% and significantly increases with age in China. The current treatment of HF is still based on Western medicines, mainly including diuretics, cardiotonic agents, vasodilators, and angiotensin-converting-enzyme inhibitors. However, the 5-year survival rate of HF is similar to that of malignant tumors [2].

Traditional Chinese Medicine (TCM) has received extensive attention and research for its long-lasting effects and fewer side effects [3]. TCM not only focuses on improving the symptoms of patients, but also pays attention to the adjustment of the patient's constitution and improve the quality of life of patients [4]. Huangqi, a classic TCM and a representative of tonic herbs with “Gan flavor” and “Wen nature,” is extensively used to strengthen “Qi” and “blood” [5]. According to clinical manifestations in TCM, HF can be classified as “chuan zheng” or “shui zhong,” which is usually related to the insufficiency of “Qi” and “blood” and always combined with phlegm-dampness and/or blood stasis. Insufficiency of “Qi” and “blood” will lead to the energy of tissues, urine and body fluid stagnation [5]. The “Gan flavor” and “Wen nature” are acknowledged as the effective treatment to reinforce “Qi” and improve “chuan zheng” or “shui zhong” syndrome. Previous studies reveal that Huangqi can improve cardiovascular function, protect myocardial cells, increase coronary blood flow, enhance myocardial contractility, and have positive inotropic effect on the heart [6, 7]. Moreover, Huangqi injection is approved by China Food and Drug Administration and has already achieved positive effect in treating HF in clinical trials [8]. However, the ingredients and targets of Huangqi in the treatment of HF have yet to be further explored.

Due to the characteristics of multicompound, multipathway, and multitarget of TCM, it is difficult to clarify the pharmacodynamic basis and mechanism of TCM. The concept of network pharmacology was first proposed by the British pharmacologist Hopkins in 2007 [9]. The molecular mechanism of drug intervention in disease is understood from a multidimensional perspective based on multidisciplinary theories such as systems biology and multidirectional pharmacology. The mechanism of TCM researched by network pharmacology is in line with the overall function of TCM, and the network pharmacology method is accurate and reliable [9]. This study used network pharmacology to construct a compound-target-disease network, which laid the foundation for further research on the mechanism of Huangqi in treating HF.

## 2. Methods

### 2.1. Collection of Compounds of Huangqi

Traditional Chinese Medicine Systems Pharmacology Database and Analysis Platform (TCMSP: http://lsp.nwu.edu.cn/tcmsp.php), one of the world's largest noncommercial TCM molecular databases, collects 499 herbs from the 2015 Chinese Pharmacopoeia and the ingredients of each herb, including 13144 molecules and 29384 compounds [10]. TCMSP provides comprehensive data on absorption, distribution, metabolism, and excretion properties for each compound. TCMSP combines pharmacodynamics, pharmacokinetics, network, omics, and system analysis to form a unified and complete system for the prediction and validation of pharmacodynamics of TCM. Oral bioavailability (OB) is one of the most important pharmacokinetic properties of oral drugs because it plays an important role in the efficiency of drug delivery to the systemic circulation. Drug-likeness (DL) is a qualitative concept designed to help optimize pharmacokinetics and drug properties, such as solubility and chemical stability [10]. In this study, the screening criteria for Huangqi were determined as OB ≥30% and DL ≥0.18 [10]. The primary molecular formulas of compounds were double-checked by PubChem (https://www.ncbi.nlm.nih.gov/pccompound) to confirm the final compounds of Huangqi.

### 2.2. Collection of Targets Related to Compounds

The targets related to compounds of Huangqi were further predicted based on the computer targeting technology developed by the TCMSP. Targets' information was obtained from TCMSP and the predicted targets were reconfirmed by DrugBank (http://www.drugbank.ca) [11]. UniProt (http://www.uniprot.org/), a database that centrally collects protein resources, is the most comprehensively and functionally annotated database of protein sequences [12]. Then, the targets were entered into UniProt, and the species selected was “*Homo sapiens*.” The repeated, nonhuman, and nonstandard targets were eliminated, and the targets related to compounds of Huangqi were finally obtained through retrieval and transformation to gene symbols.

### 2.3. Collection of Targets Related to HF

Comparative Toxicogenomics Database (CTD: http://ctdbase.org/) and GeneCards (https://www.genecards.org/) were used to select targets associated with HF. CTD is an open database that provides a centralized, integrated data of different types of molecules and toxicological data from various organisms. It integrates information about chemical-gene/protein interactions, chemical-disease, and gene-disease interactions [13]. GeneCards builds a correlation between gene and disease and provides a GiftS algorithm to further screen more relevant targets [14]. “Heart failure,” “Cardiac Failure,” “Congestive Heart Failure,” and “Myocardial Failure” performing keywords were input CTD and GeneCards to obtain targets related to HF. Finally, Draw Venn Diagram (http://bioinformatics.psb.ugent.be/Webtools/Venn/) was used to analyze the targets' intersection of HF and Huangqi.

### 2.4. Protein-Protein Interaction (PPI) Network of Targets Intersection of HF and Huangqi

The targets' intersection of HF and Huangqi was imported into the STRING 11.0 (https://string-db.org/) to perform PPI analysis. STRING, a database that predicts direct and indirect interaction and builds the network between proteins and proteins, has 9,443,763 proteins from 2031 species of organisms. A protein with a combined score >0.4 was selected to construct a interaction network [15]. The full node-node information was exported to build a PPI network of targets intersection of Huangqi and HF by using Cytoscape 3.7.1. Cytoscape is an open software for visual integration of biomolecular interaction networks with high-throughput data [16]. Therefore, overlapping targets of Huangqi and HF in the PPI network were analyzed topology parameter characterization with Network Analyzer and used MCODE to screen the important module of PPI network. The detection of dense connected regions in large-scale protein network was defined as a MCODE module and the degree of protein association in the module was scored using the following criteria: degree cutoff = 2, node score cutoff = 0.2, K-score = 2, and Max depth = 100 [15].

### 2.5. Gene Ontology (GO) Analysis of Targets Intersection of HF and Huangqi

GO is a system widely used in the field of biology for the classification of gene functions and for describing the functions of gene products [17]. Metascape (http://metascape.org/gp/index.html#/main/step1), an efficient and intuitive tool that has the Integrated Discovery and Annotation capabilities, primarily provides classical batch annotation and GO terminology enrichment interpretation to emphasize the most relevant GO terms related to an offered gene list [18]. The targets' intersection of HF and Huangqi was input into Metascape for GO analysis and the parameter selected was “*Homo sapiens*” [18].

### 2.6. Kyoto Encyclopedia of Genes and Genomes (KEGG) Pathways Analysis of Targets' Intersection of HF and Huangqi

KEGG, a knowledge base for gene function system analysis, links genomic information to higher-order functional information to obtain significantly enriched biological pathways [15]. The targets' intersection of HF and Huangqi was input into ClueGO plugin of Cytoscape 3.7.1 and the screening criterion of KEGG pathway was determined as *P* value ≤0.05 [15].

### 2.7. Network Characteristics Analysis

Network Analyzer plugin in Cytoscape 3.7.1 software was used to analyze the topology parameters of the network. This study evaluated the criticality of nodes in the network based on several parameters: (1) Average Shortest Path Length (ASPL) means the average density of the shortest path between all pairs of nodes, the smaller the ASPL, the closer the relationship between the nodes. (2) Betweenness Centrality (BC) can calculate the number of shortest paths passing through a node. The greater number of shortest paths passing through a node represents the higher BC. (3) Closeness Centrality (CC) reflects the proximity of a node to other nodes in the network. The closer a node to other nodes, the more central it is. (4) Degree represents the number of a node connected to the other nodes in a network. The greater the degree of a node, the more critical it is.

### 2.8. Molecular Docking

CB-Dock (http://cao.labshare.cn/cb-dock/) predicts the binding activities of proteins to compounds and calculates the center and size of the cavity. It is also integrated with AutoDock Vina and has been carefully optimized with a success rate of 70% [19]. PDB formats of proteins and ligand files in SDF formats of the top 10 compounds in compound-target network and the top 7 targets in PPI network and KEGG pathways were input to CB-Dock to elevate the binding activities. The style of ligand and receptor were, respectively, set as “spacefill” and “cartoon.” The color of ligand and receptor were, respectively, chosen by “element” and “chain” [19]. Digoxin, a drug commonly used in treating HF, was used for molecular docking to achieve a comparative analysis with Huangqi. The PDB formats of proteins and ligand files in SDF formats were derived from the protein database (http://www.rcsb.org) and PubChem, respectively [20].

## 3. Results

### 3.1. Compounds of Huangqi

18 compounds meeting the criteria of OB ≥30% and DL ≥0.18 and having the clear chemical structure were screened by using TCMSP database and PubChem. The 18 compounds included mairin, jaranol, hederagenin, and so on. It is worth noting that 3 compounds with outstanding pharmacological effects were also considered main active ingredients for having palmary therapeutic effect in treating HF, including astragalus polysaccharide, astragaloside IV, and cycloastragenol [21–24]. Astragalus polysaccharide and cycloastragenol are not recruited by TCMSP; thus, their detailed information was provided by PubChem. Overall, 21 compounds from Huangqi were selected for further study. The basic information of 21 compounds was shown in Table 1.

### 3.2. Compound-Target Network Construction

**Figure 1:**
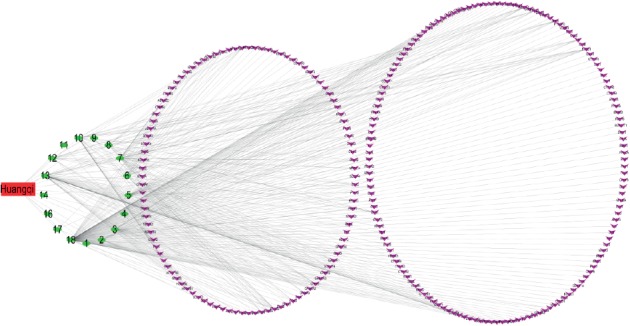
Compound-target network: the red rectangle node represents Huangqi. The green circle nodes represent compounds of Huangqi. The purple arrow nodes represent targets related to Huangqi.

The compound-target network consisted of 424 nodes, including 17 compound nodes, 407 target nodes, and 848 interacting edges. (3R)-3-(2-Hydroxy-3,4-dimethoxyphenyl)chroman-7-ol, astragalus polysaccharide, astragaloside IV, and cycloastragenol were an exception with no targets. Each edge represents the interaction between compound and target and a node with higher degree represents its importance in the network. Compound-target network shows an interaction between a compound and multiple targets and a relationship between one target and multiple compounds, which proves the synergistic effect of TCM (Figure 1). The top 10 compounds and targets details with highest degree were shown in Tables 2 and 3.

### 3.3. HF-Target and Targets' Intersection of HF and Huangqi Network Construction

**Figure 2:**
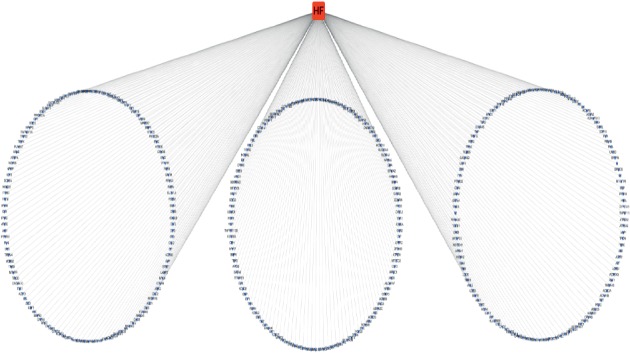
Heart failure-target network: the red rectangle node represents heart failure. The purple arrow nodes represent targets related to heart failure.

**Figure 3:**
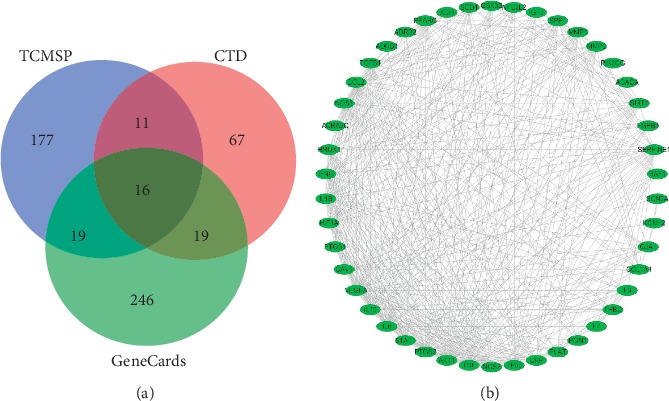
Targets' intersection of heart failure and Huangqi network. (a) The blue circle represents targets of Huangqi from TCMSP. The pink circle represents targets of heart failure from CTD. The green circle represents targets of heart failure from GeneCards. (b) Targets' intersection of heart failure and Huangqi network: the green oval nodes represent targets of targets intersection of heart failure and Huangqi.

114 targets and 10570 targets were initially screened in CTD and GeneCards, respectively. We selected the top 300 results with the highest relevance in GeneCards. 378 targets were obtained after merging and removing duplicate targets and the targets were imported into Cytoscape 3.7.1 for visualization. The targets associated with HF included HTR2B, HTR4, ADRA1A, ADORA1, ADRA1D, ADRA2C, SCNN1A, AR, and so on (Figure 2). There were 46 overlapping targets of HF and Huangqi, including PXDH, NOS2, PLAT, SOD1, PON1, IFNG, PPARG, CRP, NOS3, SERPINE1, ADRB1, TNF, VEGFA, CCL2, and so on, accounting for 11.14% of the total number of HF targets. The overlapping 46 targets represented the core and potential targets for Huangqi in treating HF (Figure 3 and Table 4).

### 3.4. PPI Network of Targets' Intersection of HF and Huangqi

**Figure 4:**
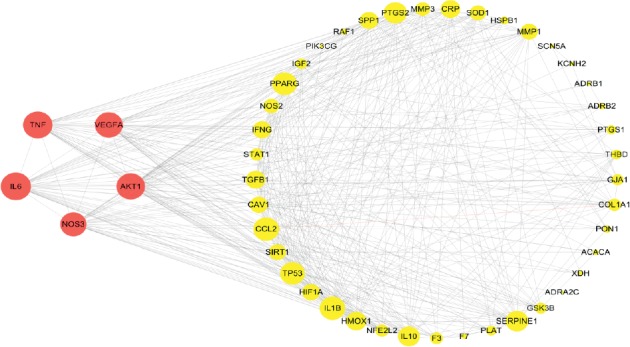
PPI network of targets intersection of heart failure and Huangqi: the red circle nodes represent the top 5 targets from PPI network of intersection of heart failure and Huangqi. The yellow circle nodes represent the other 41 targets from PPI network of intersection of heart failure and Huangqi. A larger size of a node means a greater degree.

**Figure 5:**
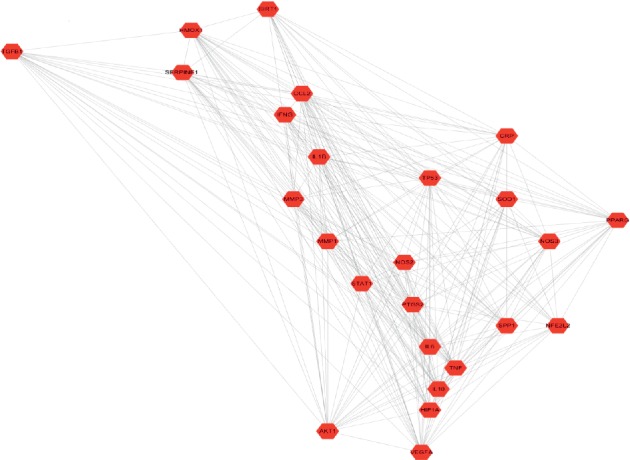
MCODE module of targets' intersection of heart failure and Huangqi: the red polygon nodes represent important targets in the most significant MCODE module from PPI network.

Nodes and edges, respectively, represented targets and associations between targets in PPI network. The size of nodes indicated the magnitude of degree and a larger size of a node meant a larger degree. A total of 46 nodes and 423 edges were involved in PPI network. The top 5 targets included IL-6 (38), TNF (37), AKT1 (36), VEGFA (35), and NOS3 (33) (Figure 4). MCODE was used to analyze the most significant module and obtained 25 core target nodes, including the top 5 targets in initial PPI network, which further proved that the importance of IL-6, TNF, AKT1, VEGFA, and NOS3 (Figure 5).

### 3.5. GO Analysis of Targets' Intersection of HF and Huangqi

**Figure 6:**
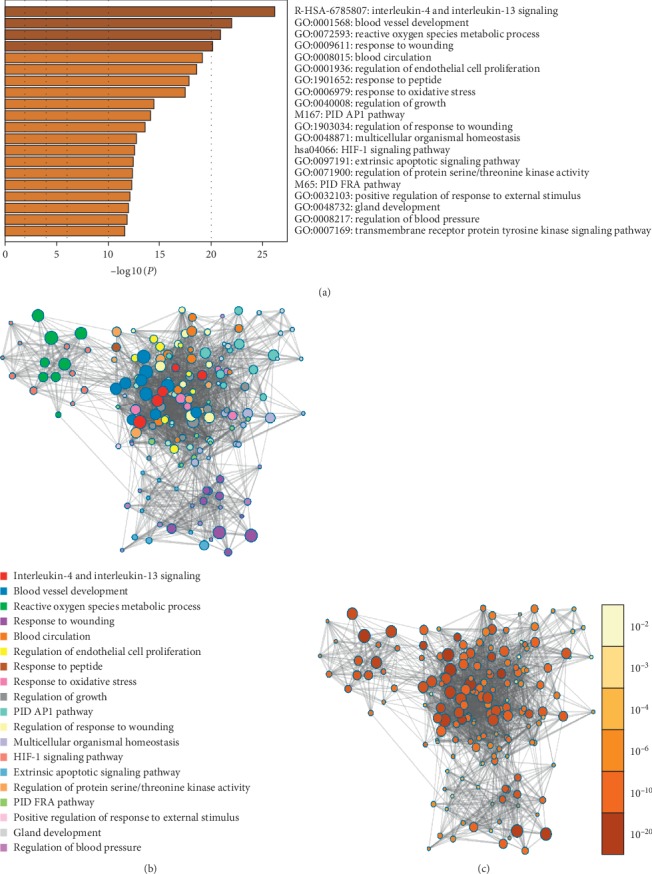
GO analysis of intersection of targets for heart failure and Huangqi. (a) The most 16 notable biological functions of targets intersection of heart failure and Huangqi. (b) A subset of enriched terms is selected and presented as a network map with terms having similarities >0.3 connected by edges. (c) Each of the nodes represents a rich term that is first colored by its cluster ID and then colored by its *P* value. The deeper the orange, the smaller the *P* value.

The most notable biological functions of targets' intersection of HF and Huangqi involved are blood vessel development, reactive oxygen species metabolic process, response to wounding, blood circulation, regulation of endothelial cell proliferation, response to peptide, response to oxidative stress, regulation of growth, regulation of response to wounding, multicellular organismal homeostasis, regulation of protein serine/threonine kinase activity, positive regulation of response to external stimulus, gland development and regulation of blood pressure, and so on. To further analyze the targets' intersection between biological processes, a subset of enriched terms was selected and presented as a network map, with terms having similarities >0.3 connected by edges. Each of these nodes represented a rich term that was first colored by its cluster ID, shown in Figure 6(b) and then colored by its *P* value. The deeper the orange, the smaller the *P* value (Figure 6) [18].

### 3.6. KEGG Pathways of Targets' Intersection of HF and Huangqi

**Figure 7:**
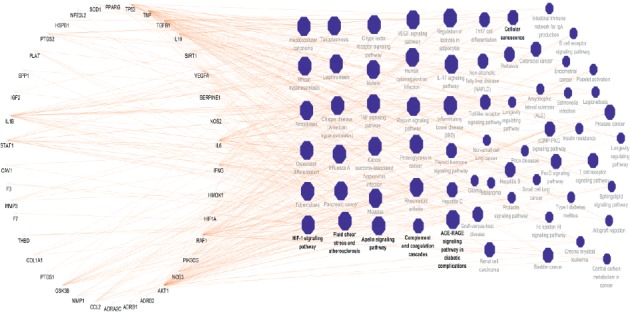
KEGG pathways of targets intersection of heart failure and Huangqi: the circle nodes represent genes enriched in KEGG pathways. The polygon nodes represent KEGG pathways of intersection of heart failure and Huangqi with *P* value ≤0.05. A larger size of a pathway means a larger degree.

It was found that overlapping targets of HF and Huangqi were involved in 65 pathways by using ClueGO analysis, including AGE-RAGE signaling pathway in diabetic complications, IL-17 signaling pathway, HIF-1 signaling pathway, apelin signaling pathway, VEGF signaling pathway, proteoglycans in cancer, TNF signaling pathway, T cell receptor signaling pathway, Toll-like receptor signaling pathway, Th17 cell differentiation, and so on (Figure 7). The top 5 signaling pathways contained AGE-RAGE signaling pathway in diabetic complications, IL-17 signaling pathway, HIF-1 signaling pathway, c-type lectin receptor signaling pathway, and relaxin signaling pathway. In addition, the top 5 targets were AKT1, TNF, RAF1, IL-6, and IL-1*β* enriched in KEGG pathways. The top 5 signaling pathways with lowest *P* value were shown in Table 5.

### 3.7. Compound-Target Docking

**Figure 8:**
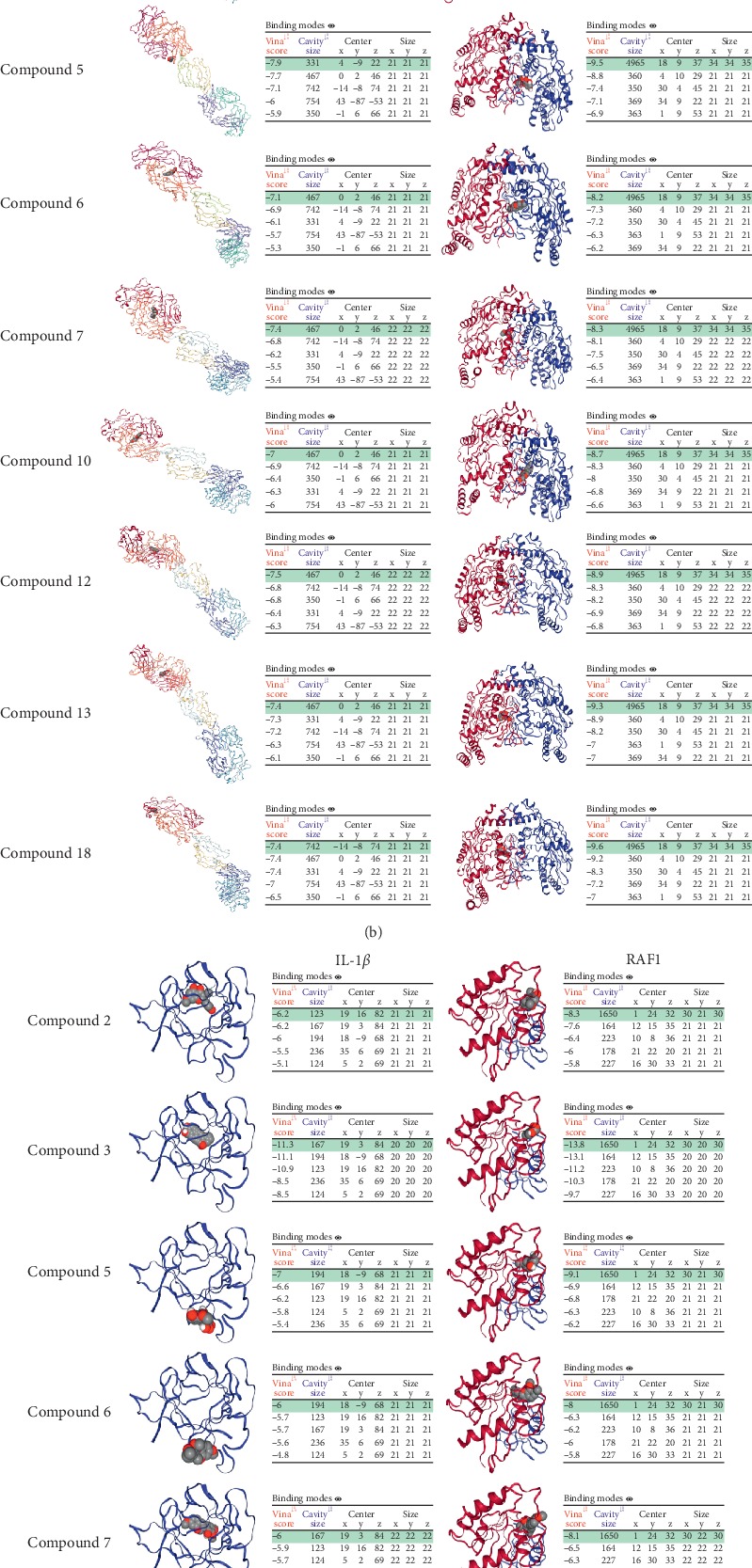
Molecular docking between compounds of Huangqi, digoxin, and targets. (a) Compound 2, compound 3, compound 5, compound 6, compound 7, compound 10, compound 12, compound 13, and compound 18 dock with IL-6 and AKT1. (b) Compound 2, compound 3, compound 5, compound 6, compound 7, compound 10, compound 12, compound 13, and compound 18 dock with VEGFA and NOS3. (c) Compound 2, compound 3, compound 5, compound 6, compound 7, compound 10, compound 12, compound 13, and compound 18 dock with IL-1*β* and RAF1. (d) Digoxin dock with IL-6, AKT1, VEGFA, NOS3, IL-1*β*, and RAF1.

The respective top 7 targets in PPI network and KEGG analysis were docked with the top 10 compounds, apart from TNF and MOL000380 with no protein PDB format and dock results, respectively. The top 5 cavities size and Vina scores were obtained from CB-Dock. The highest cavity size and the lowest binding energy (Vina score) were selected as the group representative. A spacefill and a cartoon chain represent a ligand and a protein, respectively. It is generally believed that the value of Vina score indicates that a certain binding activity between a protein and a compound. First, the more negative the binding energy (Vina score), the more stable the compound binds to the target. Second, if a cavity size is close to or bigger than the ligand, the accuracy of docking tends to increase [19]. Molecular docking results showed that the main compounds of Huangqi had good binding activities to important targets and were close to the Vina scores and cavities' size of digoxin, which is the positive control drug used to treat HF (Tables 6 and 7). Moreover, the results also showed that the Vina scores between compound 3 and IL-6, AKT1, VEGFA, NOS3, IL-1*β*, and RAF1 were higher than those of digoxin. In addition, the Vina scores between compound 13 and compound 18 and RAF1 were higher than that of digoxin. This may be because cavities' sizes of the compound 3, compound 13, and compound 18 were larger than those of digoxin (Figure 8).

## 4. Discussion

TCM has a long history of understanding the etiology and pathogenesis of HF and its effect is obvious. According to the principle of syndrome differentiation and treatment of TCM, the clinical syndromes of HF mostly rely on “Qi” deficiency and “Water” stagnation. Therefore, TCM frequently strengthens “Qi” and accelerates “Water” excretion to treat HF as the main principle. Huangqi is one of the effective drugs that can strengthen “Qi” and accelerate “Water” excretion due to its “Gan flavor” and “Wen nature” [25]. Although some studies have made preliminary research on Huangqi, the material basis and targets in treating HF have not been comprehensively clarified.

This study used network pharmacology to explore the material basis and molecular mechanism of Huangqi in treating HF. It was found that Huangqi played a potential role in improving HF by regulating multiple targets such as IL-6, AKT1, VEGFA, NOS3, IL-1*β*, and RAF1. Simultaneously, the results also showed that Huangqi may ameliorate HF through regulating multiple pathways like GE-RAGE signaling pathway in diabetic complications, IL-17 signaling pathway, HIF-1 signaling pathway, c-type lectin receptor signaling pathway, and relaxin signaling pathway. Moreover, the main compounds of Huangqi also had good binding activities to important targets compared with digoxin, which further proved that Huangqi had potential multitarget, multipathway effects on treating HF.

In this study, flavonoids, saponins, and alcohols with better OB and DL in Huangqi were screened by TCMSP and PubChem databases, which indicated that the main material basis in treating HF of Huangqi may be flavonoids, saponins, and alcohols. Flavonoids included kaempferol and quercetin, saponins contained mairin and hederagenin, and alcohols included 7-O-methylisomucronulatol. To further confirm more important material basis of Huangqi, we constructed a compound-target network; the top 10 compounds with higher degree consisted of kaempferol, quercetin, mairin, hederagenin, 7-O-methylisomucronulatol, and so on. In the compound-target network, some key compounds are highly central and are used in cardiovascular research: the levels of IL-1*β*, TNF-*α*, IL-10, IkB*α*, and NF-kB in patients with HF are reduced under the treatment of quercetin, showing that quercetin possesses the obvious anti-inflammatory properties [26]. Moreover, quercetin and isorhamnetin can inhibit the PI3K/AKT signaling pathway activation to protect heart from cardiac hypertrophy and HF [27, 28]. Study shows that calycosin and formononetin inhibit the activation of the renin-angiotensin-aldosterone system to improve the symptoms of HF [29]. Kaempferol plays a cardioprotective role by inhibiting Nrf2, NF-*κ*B, and Akt/GSK-3*β* signaling pathways to reduce apoptosis [30]. Hederagenin reduces the cascade of inflammatory responses by inhibiting IKK*β*/NF-*κ*B signaling pathway to improve coronary hemorheology and regulate endothelial dysplasia [31]. Although astragalus polysaccharide and cycloastragenol were not recruited by TCMSP, and targets corresponding to astragalus polysaccharide, cycloastragenol, and astragaloside IV were not obtained from TCMSP, they were also considered the critical compounds of Huangqi for their strong cardioprotective effect. Astragalus polysaccharide was proved to protect myocardium through reducing oxidant stress and cardiomyocyte apoptosis, inhabiting the generation of inflammatory factors like phosphorylated NF-*κ*B, IL-1*β*, IL-6, TNF-*α*, and so on, restoring normal autophagic flux [21, 32–36]. In addition, research confirms that cycloastragenol improves cardiac defect and remodeling by enhancing autophagy in myocardial cells and reduces the production of MMP-2 and MMP-9 [24]. Previous studies suggest that astragaloside IV corrects cardiac dysfunction and reduces myocardial hypertrophy by activating Nrf2/HO-1 pathway and PI3K/mTOR pathway and can promote angiogenesis [37–39]. This study recruited both identified compounds from TCMSP and the compounds with outstanding pharmacological effects. These key compounds have certain roles in anti-inflammation, antioxidation, antiapoptosis, regulating autophagy, improvement of blood perfusion, regulation of vascular endothelial function, and so on.

A total of the most 16 significant GO functional annotations were enriched in 46 overlapping targets of Huangqi and HF. More importantly, the most pivotal 7 targets from of PPI and KEGG analysis were screened using network analysis including IL-6, AKT1, VEGFA, NOS3, RAF1, IL-1*β*, and TNF. Functions of overlapping targets can be classified into steroid hormone receptor activation and regulation of heart rate and blood pressure. These functions also associated with cardiomyocyte metabolism, electrocardiogram (ECG) activity, blood perfusion, inflammation, and oxidative stress. Moreover, overlapping targets of Huangqi and HF had similar biological processes and multitarget synergistic characteristics. For example, enhancing autophagy of cardiomyocytes by inhibiting AKT1 expression and VEGFA increase contribute to improved cardiac dysfunction, angiogenesis, and remodeling [40, 41]. Elevation of IL-6 and IL-1*β* can induce cardiomyocyte apoptosis and reduce ventricular wall compliance and cause coronary microvascular endothelial inflammation [42]. Study shows that the use of nonsteroidal anti-inflammatory drugs that inhibit PTGS1 and PTGS2 increases the risk of HF as they can reduce prostaglandin E generation that can protect the heart [43]. Both ADRA2C and ADRB1 are involved in the regulation of myocardial contractility, heart rate, and blood pressure [44]. KCNH2 and SCN5A are involved in the reconstruction of myocardial scaffold proteins [45, 46]. NOS3 and NOS2 have superoxide metabolism, nitric oxide reaction, and regulation of arterial blood pressure [47]. SOD1, TGF1, TNF, RAF1, and TP53 participate in the process of cardiac remodeling [48–52]. Attenuation of mitochondrial translocation of HSPB1 resulting in damage to mitochondrial energy production capacity, elevation of HSPB1 level, or decrease in CCL2 expression helps to maintain mitochondrial function to improve systolic function [53, 54]. These indicate that biological functions of overlapping targets play important roles in treating HF.

In addition, the 65 pathways were mainly involved in regulation of inflammation and oxidative stress, immune response, fat and glucose metabolism, and regulation of angiogenesis. Diabetic cardiomyopathy is one of the mechanisms of disability and high mortality in diabetic patients and severely impairs myocardial contractile and diastolic function. The activation of AGE-RAGE signaling pathway in diabetic complications will lead to excessive production of advanced glycation end products to cause damage to cardiomyocytes and lead to HF [55]. IL-17 signaling pathway and c-type lectin receptor signaling pathway participate in immune responses [56]. Study finds that the balance of immune response is impaired accompanied with the activation of IL-17 signaling pathway in HF rats, and improving immune response imbalance to inhibit IL-17 signaling pathway and TNF*α* expression can improve cardiac function in HF rats [57]. The body can maintain the homeostasis of oxygen by activating HIF-1 signaling pathway in the hypoxic state and heart also needs a sufficient supply of oxygen to maintain effective contraction. Study confirms that HIF-1 signal pathway activation protects the heart and aorta under the hypoxic state by downregulating the TGF*β* signaling pathway in endothelial cells [58]. Moreover, lack of HIF-1 causes angiogenic disorders and myocardial fibrosis, which lead to HF [58]. The study also indicates that digoxin, the HIF-1 inhibitor, accelerates cardiac decompensation after transverse aorta constriction, which can explain that digoxin can increase cardiac contractility but not increase the survival rate of patients with HF to a certain extent [58]. Researches show that overexpression of relaxin signaling pathway has strong vasodilation, antimyocardial remodeling, anti-ischemic, antiapoptotic, and anti-inflammatory effect [17, 59–61]. Various commonly used drugs for treating HF and diabetes-related heart diseases, such as endothelin antagonists, angiotensin-converting enzyme inhibitors, and advanced glycation end products inhibitors mostly act on the above pathways. Furthermore, the most critical targets had good binding activities to main compounds, which indicated that pharmacodynamic mechanism of Huangqi had a sufficient material basis through preliminary analysis. All the results indicate that Huangqi may regulate cardiovascular inflammatory processes, participate in myocardial remodeling, enhance myocardial contractility, regulate myocardial oxidative metabolism, fat and glucose metabolism, and so on to achieve the effect on treating HF.

In conclusion, 18 compounds with better OB and DL, 3 compounds with excellent pharmacological effects, and 407 proteins were selected as targets of Huangqi. 378 targets associated with HF and 46 targets' intersection of HF and Huangqi were obtained. The compound-target network revealed that the main compounds of Huangqi had anti-inflammation, antioxidation, antiapoptosis, improvement of blood perfusion, and regulation of vascular endothelial function to exert anti-HF effect. Moreover, GO analysis prompted that 46 targets' intersection of HF and Huangqi mainly enriched in steroid hormone receptor activation, regulation of heart rate and blood pressure, cardiomyocyte metabolism, ECG activity, blood perfusion, inflammation, and oxidative stress. KEGG analysis showed that the overlapping targets were involved in AGE-RAGE signaling pathway in diabetic complications, IL-17 signaling pathway, HIF-1 signaling pathway, c-type lectin receptor signaling pathway, relaxin signaling pathway, and so on. The docking results demonstrated that main compounds exhibited good affinity to the most critical targets, especially the hederagenin. This study supplied vision for further research into protective mechanisms of Huangqi for HF and provided a method to explore the material basis of TCM or TCM formula.

## Figures and Tables

**Table 1:** Detailed information of compounds from Huangqi.

Number	Molecule ID/PubChem CID	Molecule name	Molecule weight	OB (%)	DL
1	MOL000211	Mairin	456.78	55.38	0.78
2	MOL000239	Jaranol	314.31	50.83	0.29
3	MOL000296	Hederagenin	414.79	36.91	0.75
4	MOL000033	(3S,8S,9S,10 R,13R,14S,17R)-10,13-Dimethyl-17-[(2R,5S)-5-propan-2-yloctan-2-yl]-2,3,4,7,8,9,11,12,14,15,16,17-dodecahydro-1h-cyclopenta[a]phenanthren-3-ol	428.82	36.23	0.78
5	MOL000354	Isorhamnetin	316.28	49.60	0.31
6	MOL000371	3,9-di-O-methylnissolin	314.36	53.74	0.48
7	MOL000378	7-O-methylisomucronulatol	316.38	74.69	0.30
8	MOL000380	(6aR,11aR)-9,10-dimethoxy-6a,11a-dihydro-6h-benzofurano [3,2-c] chromen-3-ol	300.33	64.26	0.42
9	MOL000387	Bifendate	418.38	31.10	0.67
10	MOL000392	Formononetin	268.28	69.67	0.21
11	MOL000398	Isoflavanone	316.33	109.99	0.30
12	MOL000417	Calycosin	284.28	47.75	0.24
13	MOL000422	Kaempferol	286.25	41.88	0.24
14	MOL000433	Antianemia factor	441.45	68.96	0.71
15	MOL000438	(3R)-3-(2-Hydroxy-3,4-dimethoxyphenyl)chroman-7-ol	302.35	67.67	0.26
16	MOL000439	Isomucronulatol-7,2′-di-O-glucosiole	626.67	49.28	0.62
17	MOL000442	1,7-Dihydroxy-3,9-dimethoxy pterocarpene	314.31	39.05	0.48
18	MOL000098	Quercetin	302.25	46.43	0.28
19	MOL000409	Astragaloside IV	785.09	22.50	0.15
20	13943286	Cycloastragenol	490.71	—	—
21	2782115	Astragalus polysaccharide	254.69	—	—

**Table 2:** Topological parameter of top 10 compounds in compound-target network.

Category	ID	ASPL	BC	CC	Degree
Compound	MOL000098	1.86	0.73	0.54	145
Compound	MOL000422	2.53	0.23	0.40	57
Compound	MOL000378	2.65	0.11	0.38	39
Compound	MOL000392	2.69	0.10	0.37	34
Compound	MOL000354	2.71	0.09	0.37	31
Compound	MOL000296	2.77	0.08	0.36	23
Compound	MOL000371	2.77	0.05	0.36	22
Compound	MOL000380	2.79	0.03	0.36	20
Compound	MOL000417	2.80	0.02	0.36	19
Compound	MOL000239	2.85	0.01	0.35	12

**Table 3:** Topological parameter of top 10 targets in compound-target network.

Category	ID	ASPL	BC	CC	Degree
Target	PTGS2	2.00	0.07	0.50	12
Target	PTGS1	2.00	0.07	0.50	11
Target	NCOA2	2.11	0.05	0.47	9
Target	AR	2.17	0.04	0.46	7
Target	DPP4	2.17	0.04	0.46	7
Target	PRSS1	3.15	0.01	0.32	7
Target	RXRA	2.40	0.03	0.42	7
Target	CHRM1	3.06	0.01	0.33	6
Target	ESR1	3.34	0.01	0.30	6
Target	ACHE	3.14	0.01	0.32	6

**Table 4:** Information from UniProt of targets' intersection of HF and Huangqi.

Number	UniProt	Target	Number	UniProt	Target	Number	UniProt	Target
1	P47989	XDH	17	P08709	F7	33	P35354	PTGS2
2	P18825	ADRA2C	18	Q12809	KCNH2	34	Q96EB6	SIRT1
3	P04792	HSPB1	19	Q03135	CAV1	35	P01584	IL1*β*
4	P09601	HMOX1	20	P37231	PPARG	36	P23219	PTGS1
5	P35228	NOS2	21	P02741	CRP	37	P13726	F3
6	P08254	MMP3	22	Q16665	HIF1A	38	P07550	ADRB2
7	P17302	GJA1	23	P29474	NOS3	39	P15692	VEGFA
8	P22301	IL10	24	Q16236	NFE2L2	40	P01137	TGF*β*1
9	Q13085	ACACA	25	P07204	THBD	41	P49841	GSK3B
10	P01344	IGF2	26	P05121	SERPINE1	42	P13500	CCL2
11	P02452	COL1A1	27	P04637	TP53	43	P03956	MMP1
12	P00750	PLAT	28	P08588	ADRB1	44	P42224	STAT1
13	P48736	PIK3CG	29	P01375	TNF	45	P05231	IL6
14	P00441	SOD1	30	P04049	RAF1	46	Q14524	SCN5A
15	P27169	PON1	31	P31749	AKT1			
16	P01579	IFNG	32	P10451	SPP1			

**Table 5:** Topological parameter of targets intersection of HF and Huangqi in KEGG pathways.

Category	Name	ASPL	BC	CC	*P* value	Degree	Enriched genes
Signaling pathway	AGE-RAGE signaling pathway in diabetic complications	2.09	0.04	0.48	4.93 × 10^−15^	14	AKT1, CCL2, COL1A1, F3, IL1*β*, IL6, NOS3, SERPINE1, STAT1, TGF*β*1, THBD, TNF, VEGFA
Signaling pathway	IL-17 signaling pathway	2.20	0.01	0.45	2.04 × 10^−9^	19	CCL2, GSK3B, IFNG, IL1*β*, IL6, MMP1, MMP3, PTGS2, TNF
Signaling pathway	HIF-1 signaling pathway	2.21	0.01	0.45	3.91 × 10^−9^	9	AKT1, HIF1A, HMOX1, IFNG, IL6, NOS2, NOS3, SERPINE1, VEGFA
Signaling pathway	C-type lectin receptor signaling pathway	1.88	0.02	0.53	1.09 × 10^−7^	31	AKT1, IL10, IL1*β*, IL6, PTGS2, RAF1, STAT1, TNF
Signaling pathway	Relaxin signaling pathway	2.23	0.01	0.45	6.17 × 10^−7^	13	AKT1, COL1A1, MMP1, NOS2, NOS3, RAF1, TGF*β*1, VEGFA

**Table 6:** Vina scores of compound-target docking.

ID	IL-6	AKT1	VEGFA	NOS3	IL-1*β*	RAF1
Compound 2	−6.3	−5.8	−7.7	−8.5	−6.2	−8.3
Compound 3	−10.7	−10	−12.3	−16.7	−11.3	−13.8
Compound 5	−6.4	−5.9	−7.9	−9.5	−7	−9.1
Compound 6	−6.1	−5.8	−7.1	−8.2	−6	−8
Compound 7	−5.5	−5.7	−7.4	−8.3	−6	−8.1
Compound 10	−6.3	−6	−7	−8.7	−6.2	−8.2
Compound 12	−6.4	−5.9	−7.5	−8.9	−6.5	−8.3
Compound 13	−6.4	−5.7	−7.4	−9.3	−7.1	−8.9
Compound 18	−6.5	−5.9	−7.4	−9.6	−7	−9
Digoxin	−7.7	−8.7	−10.4	−11.5	−9	−8.5

**Table 7:** Cavities' sizes of compound-target docking.

ID	IL-6	AKT1	VEGFA	NOS3	IL-1*β*	RAF1
Compound 2	339	138	467	4965	123	1650
Compound 3	339	134	467	4965	167	1650
Compound 5	216	134	331	4965	194	1650
Compound 6	339	134	467	4965	194	1650
Compound 7	185	134	467	4965	167	1650
Compound 10	185	134	467	4965	167	1650
Compound 12	185	134	467	4965	123	1650
Compound 13	216	134	467	4965	194	1650
Compound 18	216	134	742	4965	194	1650
Digoxin	216	134	467	363	167	227

## Data Availability

All data generated or analyzed during this study are included in this paper.
